# Yoga in Patients With Inflammatory Bowel Disease: A Narrative Review

**DOI:** 10.1093/crocol/otac014

**Published:** 2022-04-14

**Authors:** Sandeep Kaur, Adrijana D’Silva, Abdel-Aziz Shaheen, Maitreyi Raman

**Affiliations:** Department of Medicine, Cumming School of Medicine, University of Calgary, Calgary, AB T2N 4N1, Canada; Department of Medicine, Cumming School of Medicine, University of Calgary, Calgary, AB T2N 4N1, Canada; Department of Medicine, Cumming School of Medicine, University of Calgary, Calgary, AB T2N 4N1, Canada; Department of Medicine, Cumming School of Medicine, University of Calgary, Calgary, AB T2N 4N1, Canada

**Keywords:** inflammatory bowel disease, yoga, quality of life, depression, anxiety

## Abstract

Despite advancements in the treatment of inflammatory bowel disease (IBD), the global prevalence of IBD is increasing. Patients with IBD often experience a high psychosocial burden, worsening their IBD symptoms and increasing relapse, hospitalization rates, and healthcare costs, which impairs their quality of life (QoL). Evidence suggests that mind–body intervention in many chronic illnesses is effective in improving symptoms and QoL. Yoga is the most frequently used mind–body practice globally. Meta-analyses of randomized clinical trials and prospective studies have highlighted that yoga improves symptoms and QoL of patients with IBD; however, recommendations about indications for yoga as well as dose and frequency of yoga are lacking. The present narrative review aims to describe the available evidence regarding the effects of yoga on common patient-reported outcome measures in IBD, including depression, anxiety, stress, and QoL. Physicians can hence promote yoga interventions in their discussions with patients to help control these IBD-related outcome measures.

## Introduction

Inflammatory bowel disease (IBD), including Crohn’s disease (CD) and ulcerative colitis (UC), is a chronic inflammatory disorder of the gastrointestinal (GI) tract, which has a substantial impact on the mental health and quality of life (QoL).^1^ IBD is most prevalent in the Western world, where it afflicts 0.5% of the general population.^2^ However, the epidemiology of IBD is changing over both time and geography with increasing incidence in non-Westernized countries. This increase suggests that environmental factors, including psychosocial factors, may have a role in inducing or modifying disease expression.^2–4^

IBD is associated with depression, anxiety,^5^ and perceived stress,^6,7^ which are associated with poor QoL.^8,9^ Prevalence rates of anxiety and depression in patients with IBD have been reported to be as high as 30% and 40%, respectively.^10^ These comorbidities may be important determinants of disease course and relapse.^6^ A Canadian population-based study reported an association between increased risk of mortality and psychiatric conditions in patients with three immune-mediated inflammatory diseases, including IBD.^11^ These findings highlight the importance of appropriate and timely recognition and treatment of mental disorders in patients with IBD.^10^ Depression and anxiety can exacerbate IBD symptoms and increase the risk of disease flare, relapse, hospitalization rates, and healthcare costs.^5,12^ Perceived stress has been associated with symptomatic disease flares in patients with quiescent disease in prospective studies. In previous studies, including patients with IBD, perceived stress had shown a significant association with the QoL.^13^ Significantly lower overall QoL was reported compared to the general population.^13^ Furthermore, perceived stress appeared to be a marker of symptom management (self-reported pain ratings) in patients with IBD.^13^ In addition to perceived stress, anxiety and depressive symptoms correlate with GI symptoms and are associated with reduced QoL and altered illness and behavior in patients with IBD.^14^ In a systematic review with meta-analysis, compared to general populations, patients with IBD had lower generic (−0.25 [95% CI, −0.36 to −0.15]) and IBD-specific (−0.85 [95% CI, −1.14 to −0.56]) QoL.^15^ Patients may experience reduced QoL even during periods of remission.^16^ Disease severity, psychological distress, social support, and coping ^17,18^ are important determinants of QoL. Nearly 70% of patients with IBD regarded stress as an important factor influencing their disease course, and 85% reported that coping with stress effectively had a positive impact on disease symptoms.^19^ To date, the relationship between stress and objective measures of disease-related inflammation (imaging, biomarkers, and endoscopy) has not been well studied.

Although mental health affects health outcomes, psychosocial care is of low priority for some specialists.^20,21^ On one hand, physicians may not be able to recognize symptoms of depression, anxiety, and chronic stress, and perhaps lack familiarity with the identifying tools and resources (e.g., behavior health resources, validated mental health survey tools). On the other hand, patients may be hesitant to disclose their concerns out of fear of stigmatization or not identifying viable treatment options, or out of worry of lack of resources (e.g., access to appropriate providers) or financial constraints.^10,20^ Given that IBD appears to be emerging as a global disease, healthcare researchers need to reassess current treatments and resources devoted to IBD.

The American Gastroenterological Association (AGA) task force has highlighted essential screening and intervention steps gastroenterologists may use to improve mental health and enhance IBD care.^20^ However, AGA recommendations for mind–body interventions (MBI) to improve IBD care are lacking.^22^ In contrast, the European Crohn’s Colitis Organisation (ECCO) recommends cognitive behavioral therapy, meditation, relaxation, and yoga in adults with IBD to improve QoL. However, the ECCO acknowledges the scarcity of evidence on mind–body intervention to improve IBD care.^23^

## Mind–Body Interventions

According to the National Institutes of Health’s National Center for Complementary and Integrative Health, MBI are among the most popular complementary health techniques used by individuals in the general population globally,^24^ and these practices have gained acceptance in the Western world over time.^25^ Internal awareness, physical alignment, and deep breathing are used in MBI to improve individual wellness by focusing on the relationship between the mind and body. MBI are divided into two categories: (1) the motionless interventions (e.g., cognitive behavioral therapy, meditation, hypnosis, and relaxation therapy) and (2) the movement of joints and muscles (e.g., yoga, tai chi, qigong, and Pilates).^26^

MBI provide safe, inexpensive,^27^ and non-pharmacologic ways to modulate the autonomic nervous system (ANS) by decreasing stress and increasing relaxation, likely due to several mechanisms described in a later section of this review.^27,28^ Increased use of MBI may be related to ineffective prior therapies, risks involved with standard pharmacotherapy, and interest in adjunct therapies to optimize disease management.^27,29^

The effectiveness of MBI in many chronic illnesses to improve disease-related symptoms, QoL, mental health, fatigue, and sleep has been observed in cardiac diseases,^30^ chronic obstructive pulmonary disease,^24^ and cancer^31^ without any serious adverse events. These findings highlight MBI as safe and effective for symptom management in various chronic diseases yet, similar studies in patients with IBD are limited.

### Yoga

Yoga is the most frequently used MBI in the United States^32,33^ for wellness-related reasons and to treat any specific health conditions. A national health survey from the United States indicates that about 31 million adults have used yoga, and about 21 million practiced yoga in the last 12 months.^33^

Yoga, which means “yoke” or “union” in Sanskrit, is a collection of physical, mental, and spiritual disciplines originating in ancient India and aimed at controlling (“yoking”) and calming the mind.^34^ The ancient philosophy of yoga was outlined in the second century BC based on the eight components of Ashtanga yoga as described by Patanjali.^35^ The traditional Asthanga yoga practices were varied and encompassed ethical disciplines, individual observance, postures, breath control, sensory withdrawal, concentration, and meditation to attain self-realization (understanding the ultimate potential of the human being).^35^ Modernization of yoga as observed in the Western world has led to both the scientific community and general population to perceive yoga as a practice centered in physical postures with or without breathing or meditation components to enable health and manage chronic diseases.^36^ Hatha yoga is the most common form of yoga practiced in the western world and includes physical postures, breath work, and meditation. It is from Hatha yoga that several other familiar yoga styles evolved, including Iyengar, Kundalini, and Bikram, among others.^37–39^ Styles of yoga may differ in intensity, posture details, and multicomponent (postures, breathing, meditation) emphasis. In contrast, Yoga Nidra is a practice that does not utilize physical postures, but emphasizes deep relaxation and concentration focused on creating awareness.^40^

Beneficial effects of yoga have been seen in many chronic conditions, including depression,^41^ anxiety,^41^ arthritis,^42^ asthma,^43^ irritable bowel syndrome,^37^ diabetes mellitus,^44^ cardiovascular disease,^45^ and cancer^46^ with improved fitness, flexibility, strength, QoL and reduced stress, anxiety, and depression.^37,47^ Specific to IBD, a single systematic review and meta-analysis evaluated the efficacy of yoga in treating physical and psychological symptoms associated with IBD in comparison with treatment as usual or other psychotherapeutic interventions.^48^ Unfortunately, only two studies (177 patients) were included in this review (among several other mindfulness interventions). However, based on a subgroup analysis, patients receiving yoga had significant improvements in their depression and anxiety scores, without significant improvements in clinical symptoms of IBD. Here we present a narrative review summarizing the current evidence on the role of yoga in the IBD course and on patient-reported outcome measures, including depression, anxiety, stress, and QoL. Additionally, we discuss the potential mechanisms of action for the effectiveness of yoga and provide a framework for its implementation in clinical practice.

## Methods

Two electronic databases, Ovid MEDLINE and Google Scholar, were searched from May 2000 to August 2021. Search terms included the following keywords and MeSH headings: “yoga,” “yoga type,” “inflammatory bowel disease,” “Crohn’s disease,” “CD,” “ulcerative colitis,” “UC,” “quality of life,” “depression,” “anxiety,” and “stress.” Two authors (S.K. and M.R.) reviewed the titles and abstracts identified by the literature and were screened to focus on yoga and IBD published in the English language. The following inclusion criteria were applied to full-text articles: (a) study design (single-arm prospective studies and RCTs) and (b) population (adults diagnosed with IBD), intervention (yoga), comparator (not applicable), and outcomes (disease activity, anxiety, depression, stress, and QoL). Exclusion criteria included (a) case reports, conference abstracts, cross-sectional studies and (b) pediatric or adolescent populations.

## Results

The largest RCT by Cramer et al. evaluated efficacy and safety of yoga intervention in 77 patients with UC in clinical remission (defined by Rachmilewitz clinical activity index of ≤ 4 for at least 4–52 weeks prior to enrollment). Patients were randomly assigned to a yoga group (*n* = 39) for 12-week supervised sessions or a self-care written advice group (*n* = 38). Yoga practice involved a 90-minute session of Hatha yoga delivered together with Yoga Nidra. At 12 and 24 weeks, disease activity as assessed by the Clinical Activity Index (CAI) (>4 for acute flare, ≥2 and ≤ 4 for remission, and < 2 for sustained clinical remission) and HRQoL (Inflammatory Bowel Disease Questionnaire [IBDQ]) was measured. During the study, CAI decreased in the yoga group (Δ = −0.1 at 12 months and Δ = −0.8 at 24 months compared with baseline), whereas it increased in the self-care group (Δ = 0.6 at 12 months and Δ = 0.8 at 24 months compared with baseline), with a significant group difference at week 24 (Δ = −1.2; 95% CI = −0.1 to −2.3; *P* = .029). After a 6-month follow-up, patients in the yoga group reported fewer flare-ups (acute clinical flare defined as CAI > 4) compared to the self-care group (*n* = 2 in the yoga group and *n* = 5 in the self-care group; *P* = .045). For HRQoL, significant group differences in IBDQ total score favoring yoga over self-care were found at week 12 (Δ = 4.9; 95% CI = 1.9 to 8.7; *P* = .014) and week 24 (Δ = 5.7; 95% CI = 1.1 to 10.3; *P* = .015). All the subscales except social function showed an increase of three points in the yoga group. At 12 weeks, the total score increased from 143.5 ± 22.3 to 159.8 ± 32.2 in the yoga group and from 146.3 ± 24.6 to 147.1 ± 36.0 in the self-care group, resulting in a significant group difference at week 12 (Δ = 14.7; 95% CI = 2.4 to 26.9; *P* = .018). Group differences were sustained at week 24 (Δ = 16.4; 95% CI = 2.5 to 30.3; *P* = .022). Yoga intervention was found to be safe as non-serious or serious adverse events did not differ between groups (*P* = .7). Non-serious adverse events in the yoga group included temporary, transient musculoskeletal pain (5 cases), acute flare (1 case), and increase in UC symptoms without flare (1 case). Serious adverse events in this group included two cases of acute flares leading to hospitalization (2 cases) and newly diagnosed colorectal cancer (1 case). One case of spinal canal stenosis was reported in the self-care group. This study concluded that the yoga intervention improved QoL in patients with UC compared with written self-care advice.^32^ In a secondary analysis of Cramer et al 2017, HRQoL and disease activity were found to be substantially associated with perceived stress at week 24. The reported effect of yoga on QoL (β = 16.23; 95% CI = 6.73 to 28.40) and disease activity (β = −0.28; 95% CI = −0.56 to −0.06) at week 12 were fully mediated at week 24.^49^

Another RCT by Sharma et al. explored the efficacy of an 8-week supervised yoga intervention (physical postures, pranayama, and meditation, 1 hour/day in addition to standard medical therapy) compared with standard medical treatment alone among 60 UC and 40 CD patients in clinical remission. Although clinical disease activity was not measured using a validated tool, self-reported presence or absence of clinical symptoms (tenesmus, intestinal colic, peri-anal pain, arthralgia, and anorexia) was evaluated using a diary once daily before going to bed. In addition to clinical symptoms assessment, anxiety levels were measured by the State-Trait Anxiety Inventory. A significant reduction in state anxiety (Δ = −6.08; *P* = .01) and trait anxiety (Δ = −8.24; *P* = .001) was noticed at the end of the 8-week intervention in the patients with UC practicing yoga compared with the controls without any adverse events. However, in patients with CD, anxiety levels post-intervention were unchanged (*P* > .05). It was explained that patients with CD might require prolonged interventions and individualized therapy. No significant difference was observed in clinical symptoms between the groups at 4 weeks; however, a higher number of patients in the control group (*n* = 14) reported intestinal colic pain at 8 weeks compared to the yoga group (*n *= 5; *P* < .05).^50^

In a small prospective study by Kaur et al.,^51^ nine patients with IBD, both clinically active and in remission, completed an 8-week pilot study followed by semi-structured interviews to assess the acceptability (assessed using adherence and safety) and effectiveness of an integrated yoga program. A certified yoga facilitator delivered the intervention in person for 30 minutes weekly with a requirement for daily home practice. During the first 2-hour session, students received a live introduction and demonstration of the various components used in the yoga intervention. The intervention included Hatha yoga (postures, breathing practices, breath watching, and chanting) and foot massage with lavender oil before bedtime. Validated survey tools examining anxiety (Generalized Anxiety Disorder-7 [GAD-7]), depression (Patient Health Questionnaire-9 [PHQ-9]), perceived stress (Perceived Stress Scale [PSS-10]), sleep quality (Pittsburgh Sleep Quality Index [PSQI]), and HRQoL (12-Item Short-Form Health Survey [SF-12]) were used. Participants were instructed to complete the same routines every day for 8 weeks, with routines documented weekly in a diary. Class attendance data and a weekly home practice log were used to measure adherence. High adherence to the intervention was observed, with participants reporting an average of 5.4 days of practice each week. The interviews identified three main themes where the participants reported feeling the intervention was beneficial; however, they felt guilty about not completing daily practices, which decreased confidence and intention to continue with the practice. There was a decrease in the PHQ-9 depression score (Δ = −4.4) indicating improvement in depression symptoms. A decrease in the GAD-7 anxiety score (Δ = −1.2) and PSS-10 score (Δ = −0.1) was observed. An improvement in mental health score was observed (Δ = 5.2 in physical health component score and Δ = 6.4 in mental health component score) suggesting an improvement in HR QoL after 8 weeks of yoga intervention.^51^

The published literature reporting on yoga as an intervention in patients with IBD is very limited. Nevertheless, from the available literature, summarized (Table 1) in this present narrative review, it can be inferred that yoga is an effective therapy to improve QoL and mental health by decreasing anxiety and stress for patients with IBD. Possibly yoga may have a role to maintain clinical remission; however, the published studies are limited either by a short duration of follow-up or lack of validated tools to draw firm conclusions.

**Table 1.** Summary of the characteristic of the included studies

Author; year	Study type	N/Type of patients	Study groups	Intervention and control details	Modality	Length	PROM	Findings	Conclusion
Cramer et al; 2017	RCT	77 UC patients in clinical remission	(i) Yoga (*n* = 39) (ii) Self-care written advice (*n* = 38)	(i) 90-minute session of Hatha yoga delivered together with yoga nidra; 12 sessions (ii) Written Self-care advised	Supervised	24 weeks	(i) Disease activity assessed by CAI (ii) HR QoL assessed using IBDQ	(i) CAI decreased in yoga group, increased in self-care group both at 12 and 24 months (ii) Significant group differences in IBDQ total score favoring yoga over self-care (p = 0.015)	Yoga can be considered as a safe and effective ancillary intervention for patients with UC and impaired QoL.
Sharma et al; 2015	RCT	60 UC and 40 CD patients in clinical remission	(i) Yoga (*n* = 50; 30 UC, 20 CD) (ii) Standard medical treatment (*n* = 50; 30 UC, 20 CD)	(i) Eight-week yoga; physical postures, pranayama, and meditation), 1- hour/day in addition to standard medical therapy (ii) Standard medical treatment	Supervised	8 weeks	(i) Anxiety levels assessed by State-Trait Anxiety Inventory (ii) Clinical symptoms	(i) A significant reduction in state anxiety (Δ = -6.08; p = 0.01) and trait anxiety (Δ = -8.24; p = 0.001) at the end of the 8-week in the patients with UC with yoga without any AE; in CD patients, anxiety levels was unchanged (p > 0.05). (ii) No significant difference in clinical symptoms between the groups at 4 weeks, 5 patients with UC out of 25 in the yoga group reported arthralgia compared to control (6 out of 26; p > 0.05), and a higher number of patients in the control group (n = 14) reported intestinal colic pain compared to yoga group (n = 5; p < 0.05)	A simplified yoga-based regimen is a safe & effective complementary clinical treatment modality for patients with IBD during the clinical remission phase
Koch et al; 2020	Secondary analysis	77 UC patients in clinical remission	(i) Yoga (*n* = 39) (ii) Self-care written advice (*n* = 38)	(i) 90-minute session of Hatha yoga delivered together with yoga nidra; 12 sessions (ii) Written Self-care advised	Supervised	24 weeks	(i) Disease activity assessed by CAI (ii) HR QoL assessed using IBDQ	The reported effect of yoga on QoL and disease activity at week 12 were fully mediated at week 24.	HR QoL & disease activity were found to be substantially associated with perceived stress
Kaur et al; 2021	Prospective study	6 CD and 3 UC patients both active and in clinical remission		30-minute session/week; Hatha yoga-based Yoga Namaskar (10 min), breathing practices or “Nadi Shuddhi” (5 min), meditation consisting of AUM chanting (5 min) and breath watching (3–5 min), and self-foot massage with lavender oil prior to bedtime	Supervised	8 Week	(i) Primary: Adherence (ii) Secondary: depression (PHQ-9), anxiety (GAD-7), HR QoL (SF-12), perceived stress (PSS-10), & sleep quality (PSQI)	High adherence to the intervention was observed (average of 5.4 days of practice/week). There was a decrease on the PHQ-9 depression score (Δ = −4.4), GAD-7 anxiety score (Δ = −1.2) and PSS-10 score (Δ = −0.1). An improvement in mental health score was observed (Δ = 5.2 in physical health component score and Δ = 6.4 in mental health component score) suggesting an improvement in HR QoL after 8 weeks of yoga intervention.	Study supports the acceptability of an integrated yoga practice, including postures, breathing, meditation plus an aromatherapy and lavender oil intervention in IBD patients.

RCT: Randomized Clinical Trials; UC: Ulcerative Colitis; CD: Crohn’s Disease; PHQ-9: Patient Health Questionnaire-9; GAD-7: Generalized Anxiety Disorder-7; SF-12: 12-Item Short Form Health Survey; PSS-10: Perceived Stress Scale; PSQI: Pittsburgh Sleep Quality Index; CAI: Clinical Activity Index; HR QoL: Health-related quality of life; IBDQ: Inflammatory Bowel Disease Questionnaire; Δ: Represent change; IBD: Inflammatory bowel disease; QoL: Quality of life; AE: Adverse events.

### Mechanisms of Yoga

**Figure 1.**
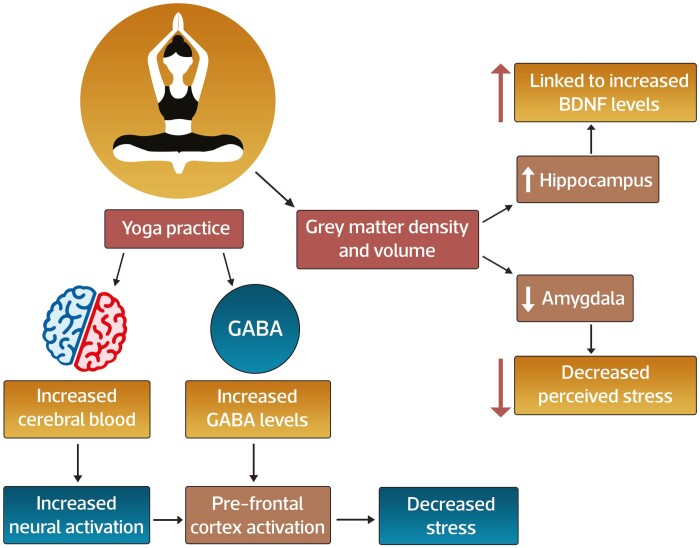
Effect of yoga practice on central nervous system structure and function. The central mechanisms of action for the therapeutic effects of yoga involve changes in grey matter morphology, enhanced cerebral blood flow, and cerebral activity, resulting in altered neurotransmitter (GABA, BDNF) expression. The mechanism involves increasing grey matter density in yoga practitioners with its effects observed in the hippocampus and amygdala. The effects of hippocampal are linked to increased BDNF levels, a marker for neuroplasticity, whereas a decline in amygdala volume is associated with decreased perceived stress among yoga practitioners. Yoga shows its effects on cerebral blood, with many studies demonstrating increased neural activation in the pre-frontal cortex during yoga practice. Following yoga practice, an increase in GABA levels can be observed associated with decreased stress and may be a consequence of pre-frontal cortex activation.

Despite the increasing use of yoga as a therapeutic intervention for chronic diseases, its neurobiological effects are still incompletely understood. Proposed mechanisms of action for yoga effects on stress, mental health, and inflammation are varied and include effects on structural and functional brain changes and psycho-neuro-immunobiological effects (Figure 1). Neuroimaging findings^52^ in yoga practitioners compared to controls identify decreases in density and volume of the amygdala and increased grey matter volume and density of the hippocampus. The hippocampal effects could be linked to increased brain-derived neurotrophic factor (BDNF) levels which are of high concentration in this region and are^53^ a marker for neuroplasticity, while amygdala volume decline has been associated with decreased perceived stress among yoga practitioners.^54^ Yoga affects cerebral blood, with several studies demonstrating increased neural activation in the pre-frontal cortex during yoga practice. Increased gamma-aminobutyric acid (GABA)^55,56^ levels were observed following yoga practice and were associated with decreased stress, potentially as a consequence of pre-frontal cortex activation. Collectively, a unifying neurobiological model to explain the central mechanisms of action for the therapeutic effects of yoga may involve changes in grey matter morphology, enhanced cerebral blood flow, and cerebral activity, resulting in altered neurotransmitter (GABA, BDNF) expression. Furthermore, the established effects of yoga to augment the parasympathetic tone may occur through deep and slow breathing^47,52^ and by activated baroreceptors through different yoga poses, leading to its anxiety and stress-relieving effects.

Small studies have demonstrated the effects of yoga on peripheral inflammatory markers, enhancement of cell-mediated and mucosal immunity, and even effects on gene expression associated with interferon regulation and signaling, vascular permeability, and neutrophil recruitment. In addition, changes in stress-related gene expression can counteract the effects of stress on patients with IBD.^57^ However, high-quality studies are needed to explore these proposed peripheral mechanisms, and these remain largely hypothetical at present. Overall, there may be several mechanisms by which yoga may influence the course of disease in IBD, with robust evidence pointing towards its impact on stress and psychological processes. Future research is warranted to better understand the mechanisms by which MBI, such as yoga, leads to the multitude of psychological and physical changes in IBD.

### Practical Application of Yoga for Clinicians

The evidence base for yoga as a medical therapy for a wide variety of chronic physical and psychological conditions is accumulating. Yoga engagement in North America is high, with over 12% of adults and children^58^ in the United States having used yoga and/or deep breathing exercises over 12 months. Gastroenterologists should be aware of the spectrum of the effectiveness of yoga, as several patient-reported outcome measures can be enhanced through yoga practice. Further, gastroenterologists should be aware that patients are likely to be accepting of yoga as a recommended therapy.

While there are no specific guidelines about patient selection, yogic components, yoga dosage or even indications, the following framework may be considered:

- Is the patient interested in yoga, breathing or meditation, and are they willing to implement behavioral changes to engage with yoga practice?
- Is yoga accessible in terms of time, location, and cost?
- Does the patient have impaired QoL, clinically significant depression, anxiety or perceived stress that is either not managed or incompletely managed with prescription mediations?
- Does the patient report poor sleep quality or fatigue?

An affirmative response to the above points may serve as a starting point to share yoga and outcome-focused education with the patient. Ideally, patients should identify a certified yoga instructor or facilitator with experience in chronic diseases, plan to engage with yoga postures, breathing exercises, and mediation five days weekly for 6 weeks as a frequent multimodal practice has been associated with the greatest benefit. This dosing recommendation comes from a recent RCT involving 101 people with depression and/or anxiety symptoms (compared with a waitlist control group) found practicing yoga for 4.8 days per week, 29 min per session for 6 weeks was sufficient in improving depression and anxiety symptoms (Depression Anxiety Stress Scale [DASS-21]), along with decrease distress (Kessler Psychological Distress Scale), negative experiences (Scale of Positive and Negative Experience), and increased resilience (Connor-Davidson Resilience Scale). All benefits remained 6 weeks post-intervention..^59^ This dosing (5 days a week for 25–30 min per day) was achievable for most participants. Studies such as these provide a starting point for future investigations on yoga interventions with patients with IBD to determine optimal dosing for this population. While the evidence base for virtually delivered multi-modal yoga intervention is low, virtual CBT interventions for patients with IBD are promising.^60^ Future studies evaluating the effectiveness of virtually delivered yoga interventions should be considered to improve access and decrease barriers to participation.

## Strength and Limitations

The only published systematic review and meta-analysis reported the effectiveness of two yoga RCTs for patients with IBD on mental health and QoL. Lack of mechanistic rationale and discussion on positioning yoga as a therapeutic offering were the few limitations of this review. While remission duration was explored, short follow-up times limited meaningful interpretation. Our work presents an updated review of this literature to include these two RCTs, a secondary analysis, and a prospective study. In addition, we integrate proposed mechanisms for the effectiveness of yoga and provide a framework for its implementation into clinical practice. Limitations of the published literature include few studies with an RCT design, heterogeneous interventions, variable sample sizes, differences in intervention components, variable baseline IBD activity, and outcome measures. The yoga interventions in the RCTs included both physical postures, breath work, and meditation. However, the duration of total practice, duration of sub-components, and intensity of physical postures differed. Future research will benefit from a mechanism-driven approach to defining an optimal yoga intervention and testing its effectiveness through standardized delivery.

Future directions would include robust epidemiological or clinical trial data to better understand the IBD-specific outcomes that can be influenced by yoga practice (induction and maintenance of clinical remission, and effects on gut inflammation) and the most effective components and duration of yoga practice to achieve desirable outcomes. Determining patient attitudes and beliefs towards yoga, understanding its accessibility, barriers and facilitators to yoga engagement, and the role of virtually delivered interventions can provide insight into optimal therapeutic positioning.

## Conclusion

This narrative review summarizes the published literature in the field and offers a practical implementation strategy for physicians to apply in the clinic setting. Further, the potential mechanisms for yoga effectiveness are reviewed in detail. These mechanisms may provide a foundation from which to design specific interventions in the future. Small studies suggest promise for the beneficial outcomes of yoga, in particular depression, anxiety, and QoL in patients with IBD. In support of the experience within IBD, the broader literature would confirm the mental health and QoL benefits observed in this context. The proposed framework for yoga implementation into clinical practice may be considered while this body of literature evolves.

## Data Availability

We do not have any data as this is a review.
